# Evaluation of target and cardiac position during visually monitored deep inspiration breath‐hold for breast radiotherapy

**DOI:** 10.1120/jacmp.v17i4.6188

**Published:** 2016-07-08

**Authors:** Leigh Conroy, Rosanna Yeung, Elizabeth Watt, Sarah Quirk, Karen Long, Alana Hudson, Tien Phan, Wendy L. Smith

**Affiliations:** ^1^ Department of Medical Physics Tom Baker Cancer Centre Calgary; ^2^ Department of Physics and Astronomy University of Calgary Calgary; ^3^ Department of Oncology University of Calgary Calgary; ^4^ Department of Radiation Therapy Tom Baker Cancer Centre Calgary AB Canada

**Keywords:** DIBH, breast, MV cine, respiratory motion

## Abstract

A low‐resource visually monitored deep inspiration breath‐hold (VM‐DIBH) technique was successfully implemented in our clinic to reduce cardiac dose in left‐sided breast radiotherapy. In this study, we retrospectively characterized the chest wall and heart positioning accuracy of VM‐DIBH using cine portal images from 42 patients. Central chest wall position from field edge and in‐field maximum heart distance (MHD) were manually measured on cine images and compared to the planned positions based on the digitally reconstructed radiographs (DRRs). An in‐house program was designed to measure left anterior descending artery (LAD) and chest wall separation on the planning DIBH CT scan with respect to breath‐hold level (BHL) during simulation to determine a minimum BHL for VM‐DIBH eligibility. Systematic and random setup uncertainties of 3.0 mm and 2.6 mm, respectively, were found for VM‐DIBH treatment from the chest wall measurements. Intrabeam breath‐hold stability was found to be good, with over 96% of delivered fields within 3 mm. Average treatment MHD was significantly larger for those patients where some of the heart was planned in the field compared to patients whose heart was completely shielded in the plan (*p* < 0.001). No evidence for a minimum BHL was found, suggesting that all patients who can tolerate DIBH may yield a benefit from it.

PACS number(s): 87.53.Jw, 87.53.Kn, 87.55.D‐

PACS number(s): 87.53.Jw, 87.53.Kn, 87.55.D-

## I. INTRODUCTION

Adjuvant whole-breast radiation therapy (WBRT) following surgery has been shown to significantly reduce locoregional recurrence and improve survival for breast cancer patients.[(1,2)] However, WBRT has also been shown to increase the risk of long-term cardiac disease,[(3)] particularly in left-sided breast cancer patients.[(4)] There is some evidence that the left anterior descending artery (LAD) may be a critical cardiac structure in RT-induced ischemic heart disease risk.[(5)] A recent study has shown that addition of regional nodal irradiation (RNI) including the internal mammary chain (IMC) nodes leads to a reduced rate of breast cancer recurrence at 10 years for node positive or high-risk node negative patients;[(6)] however, RNI of the IMCs can result in even higher cardiac doses than standard WBRT.[(4,7)]

[a] Corresponding author: Leigh Conroy, Department of Medical Physics, Tom Baker Cancer Centre, 1331 29 St. NW, Calgary, AB T2N 4N2, Canada; phone: (403) 521 3902; fax: (403) 521 3327; email: laconroy@ucalgary.ca

Deep inspiration breath-hold (DIBH) is a commonly used method to reduce heart dose during left-sided breast radiotherapy. There are numerous commercial methods of implementing DIBH including spirometry,(8) external markers,(9) and optical surface monitoring;(10) however, wide-scale implementation of these technologies can be resource intensive. It has been estimated that breast cancer radiotherapy represents approximately one-third of all work in radiation oncology,(11) and slightly over half of breast cancer patients have left-sided breast cancer.(12) Thus, the implementation of DIBH for all left-sided breast cancer patients can represent a significant resource and infrastructure challenge.

In response to these constraints, some clinics have implemented breath-hold techniques where existing infrastructure is used to manually gate the radiation beam during breath-hold.(13,14) We have implemented a low-resource visually monitored DIBH (VM-DIBH) technique for all eligible left-sided breast cancer patients. The technique requires no additional equipment and minor changes in clinical workflow. Here we describe the clinical protocol, verify and characterize the target positioning reproducibility, and report on the incidence of cardiac irradiation when using VM-DIBH based on MV cine portal images collected during treatment. We also aim to find evidence for a recommended minimum breath-hold level for VM-DIBH.

## II. MATERIALS AND METHODS

### A. Visually monitored DIBH

The VM-DIBH technique for left-sided breast cancer patients was implemented in our clinic using couch top to midaxillary measurements, in-room lasers and cameras, and audio coaching for manual beam gating during DIBH starting in January 2013.

#### *A.1 Patient eligibility*

All left-sided breast cancer patients able to maintain DIBH for 20 s or longer are considered eligible for DIBH at our center. There are no minimum breath-hold level, age, or treatment intent exclusion criteria. For the purposes of this study, males and right-sided DIBH patients were excluded. Patients received a DIBH information sheet at their radiation oncology consultation prior to their CT simulation.

#### *A.2 CT simulation*

Immediately prior to simulation, radiation therapists verbally explained the DIBH procedure to patients. CT simulation and treatment were performed in the supine position with both arms raised via wing-board immobilization. All CT scans were performed using a Philips Brilliance Big Bore CT (Philips Medical Systems, Eindhoven, The Netherlands). Prior to the CT scan, the distance from the couch top to the left midaxillary line BB representing the CT reference was measured and recorded for free-breathing (FB) and DIBH (Fig. 1, top pictures). The difference between the DIBH and FB measurements is defined as the breath-hold level (BHL). DIBH reproducibility was established by repeating the measurement until three stable consecutive measurements were achieved. The superior–inferior (SI) displacement of the anterior CT reference BB was also recorded (Fig. 1, bottom pictures). Based on results from a previous study,(7) patients with intent to treat regional nodes including IMCs had only a DIBH CT scan. These patients were treated in FB only if they were unable to perform reproducible DIBH as evaluated by the simulator radiation therapists. Patients receiving WBRT alone had a FB scan first, where radiation therapists evaluated the separation between the heart and the chest wall. This measurement is used *a priori* to determine if there is sufficient separation between the heart and the chest wall before the tangents are chosen (Fig. 2(b)). If this separation was greater than 5 mm on all slices, the patient was treated in FB (and not included in this study); otherwise, a DIBH scan was also performed and the radiation oncologist later evaluated the two scans to determine whether the patient would be treated in FB or DIBH. The DIBH CT scan was taken with monitoring bellows to ensure DIBH stability during the scan. Bellows were only used at simulation, and were unavailable on the treatment units.

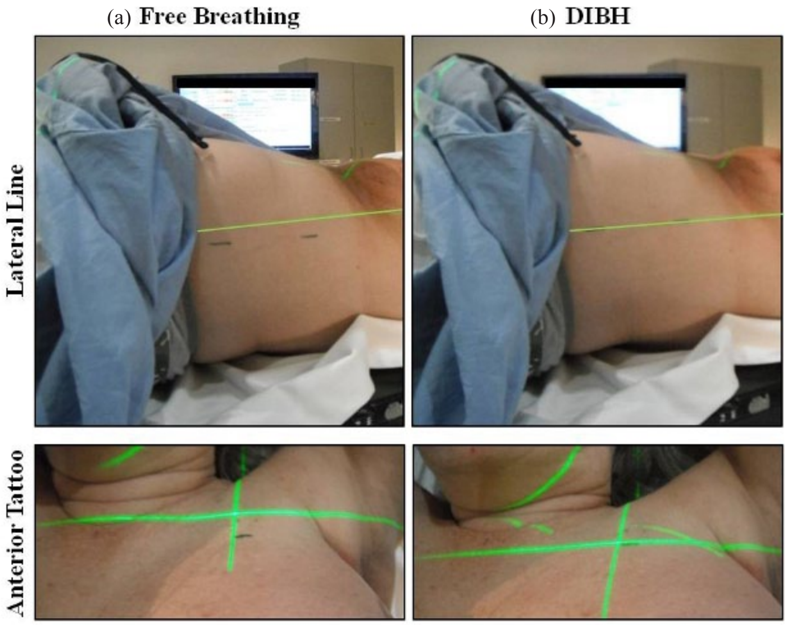

Fig. 1. Visually monitored DIBH setup technique: (a) free-breathing; (b) DIBH. Lateral lasers in the photo have been enhanced to improve visibility.

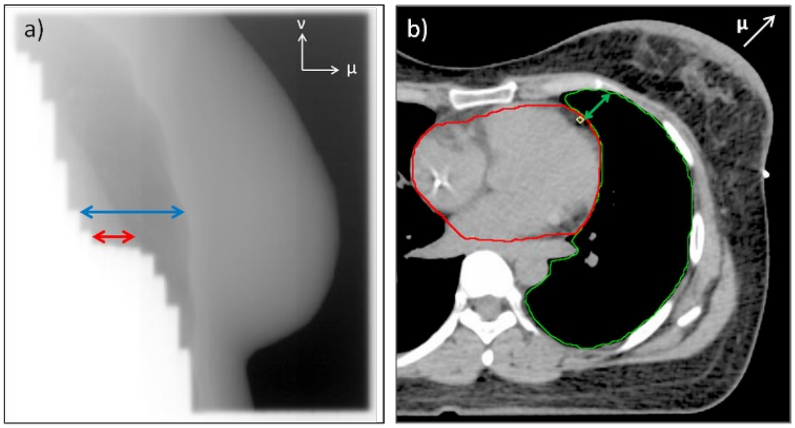

Fig. 2. (a) Cine image with chest wall position measurement at the center of the field (blue arrow) and maximum heart distance measurement (red arrow) in the μ direction; (b) axial slice of DIBH scan showing the heart (red), left lung (green), and LAD (yellow) contours, as well as the mid-LAD chest wall separation measurement (green arrow). An approximate μ direction is shown, as it is dependent on the tangent field angle.

### A.3 Treatment planning

3D conformal radiotherapy (3D CRT) treatment plans were developed on the DIBH CT scan for all patients included in this study. Tangential enhanced dynamic wedge fields were defined to encompass the whole breast or chest wall target using anatomical landmarks. Standard tangents were used for breast-only irradiation and modified wide tangents were used for patients receiving IMC nodal irradiation.(6,15) Axillary/supraclavicular nodes were treated with a single direct field or parallel opposed fields using a single isocenter technique. Single-energy 6 MV plans were used for most patients, and mixed energies (6 MV and 10 or 15 MV) were used as appropriate to reduce hot spots.

Whole-breast dose fractionation was typically 42.5 Gy in 16 fractions. For patients receiving additional RNI, dose fractionation ranged from 40−50.4 Gy in 16–28 fractions to the breast or chest wall and 37.5−45 Gy in 16–25 fractions to the regional nodes. Dose calculations were performed using Eclipse AAA version 8.9.08 (Varian Medical Systems, Palo Alto, CA) with heterogeneity corrections on. The heart and LAD were contoured according to previously published guidelines.(16)

### A.4 Treatment and imaging

On the first day of treatment, patients were aligned to their CT-simulation tattoos and shifts were performed in free-breathing. Patients were coached to perform DIBH until the anterior displacement of the left lateral tattoo matched that recorded at the simulator. A line was drawn on the patient's side where the lateral laser fell during DIBH, as illustrated in Fig. 1 (top pictures). A second line was also drawn where the crosshairs fell on the patient's chest during DIBH (Fig. 1, bottom pictures), which corresponded to the SI location of the isocenter in DIBH.

When bolus was required, it was secured in the correct position using tape. The anterior edge of the bolus was placed at the field match line in DIBH, thus the bolus remained on the patient for all treatment fields. The horizontal marker line used to guide manual gating was drawn on the cellophane wrap of the bolus and was extended inferiorly past the bolus onto the patient's skin. Treatment therapists zoomed the cameras to the marker line at the bolus/skin interface, so that if the bolus moved at any point during treatment, the line became discontinuous and the treatment was stopped to readjust the bolus.

Following setup, radiation therapists zoomed the in-room cameras to the skin line and verbally coached the patient over the intercom to perform DIBH. Therapists manually turned the beam on for each field when the skin line was coincident with the lateral laser. If the gantry blocked the camera view of the skin line in the lateral tangent field position, a second line was drawn on the patient's right side. Patients were usually treated with one breath-hold per field. A clip-on tag was placed on the treatment unit beam-on key to provide a tactile reminder for radiation therapists to ask the patient to perform DIBH.

At the end of the first day of treatment, radiation therapists tattooed a free-breathing mark on the patient's chest (anterior tattoo). The distance from the anterior tattoo to the DIBH line located inferiorly on the patient's chest was measured and recorded. This measurement was used for patient setup on subsequent days. On the following treatment days, the couch was shifted inferiorly by the DIBH displacement from the anterior tattoo to redraw the anterior DIBH line. The lateral line was then remarked during DIBH based on the anterior line DIBH position, and manual gating was performed using the line on the patient's side.

Pretreatment anterior–posterior (AP) and medial tangent field MV portal images were taken during breath-hold for the first three days and once weekly thereafter. Shifts were performed with a corrective action level of 5 mm, and when executed, necessitated redrawing the lines on the patient. During implementation of this technique, the first day of treatment was allotted 45 min, and the following treatments were allotted 30 min as compared to free-breathing patients who were respectively scheduled in 30 and 15 min time-slots. Some of the setup processes for VM-DIBH have been refined and streamlined since implementation; the reader is referred to the discussion section for more details.

## B. Cine imaging and analysis

Continuous MV portal images (cines) were taken once weekly during the medial tangent field(s) on nonimaging days. Depending on the configuration of the treatment unit and the beam energy, individual cine images were formed from an average of 4–8 frames sampled at a rate of 7–15 frames per s resulting in ~ 1–4 cine images per s. Cine images were retrospectively evaluated for this study, and were not used to make treatment decisions. Wilcoxon Rank-Sum with significance level 0.05 was used for all statistical testing.

### *B.1 Chest wall position measurements*

For chest wall positioning measurements, the distance from the field edge to the chest wall at the center of the field was manually measured on the cine images in 2 s intervals in the μ direction (Fig. 2(a)). Because the images were taken at an oblique angle, the μ direction was a linear combination of the lateral (LR) and AP directions. The same measurements were made on the digitally reconstructed radiographs (DRRs) for comparison.

Chest wall setup uncertainty was quantified by subtracting the DRR chest wall measurement from the measurement made on each 2 s interval cine image. After evaluating all cine images, the maximum difference from the DRR measurement was taken as the positioning error: $D_{SETUP} = \max_{i=1,\ldots,N}[D_{cine,i} - D_{DRR}]$. Intrabeam chest wall motion was measured by subtracting the first cine chest wall measurement from each of the subsequent cine image measurements and taking the maximum difference for each measured beam: $D_{BEAM} = \max_{i=2,\ldots,N}[D_{Cine,i} - D_{Cine,1}]$. Cine images where the wedges blocked the chest wall were not used. These values were used to compute the interfraction population mean (M), as well as systematic (Σ) and random (σ) uncertainties.[17] Chest wall positioning uncertainties in patients that underwent breast-conserving surgery were compared to those who underwent mastectomy to determine if chest wall positioning uncertainty was surgery-type dependent.

### *B.2 Maximum heart distance measurements*

The primary motivation to implement DIBH is to reduce cardiac dose; therefore, we also report on incidence and magnitude of cardiac irradiation, both planned and unplanned. All cine images were reviewed for an anterior pericardial shadow. When the heart was visible in the field, the largest maximum heart distance (MHD) at any field position during the cine video was manually measured from the field edge to the edge of the pericardial shadow (Fig. 2(a)). On the DRRs, the MHD was taken from the edge of the field to the heart contour, which included the fatty tissue of the pericardium as this region contains cardiac vessels (including the LAD).[16] If the heart contour was outside the field or at the field edge the patient was classified "Heart Planned out of Field"; if any portion of the heart contour was inside the field, the patient was classified as "Heart Planned in Field." The correlation between chest wall positioning error and MHD difference between treatment and planning was investigated to determine if chest wall displacement from the planned position could predict for unplanned MHD during treatment.

## C. Correlation between breath-hold level and LAD chest wall separation

An in-house program was designed to extract the minimum distance from the LAD to the chest wall using the Computational Environment for Radiotherapy Research (CERR), version 4.6.[18] CERR is an open-source platform for radiotherapy treatment planning that was installed in MATLAB, R2014a (MathWorks, Inc., Natick, MA). The chest wall was not routinely contoured for patients in this study; therefore the lateral edge of the left lung was used as a surrogate. An automated measurement was made between the left-most point of the LAD and the lateral edge of the lung at a 45° angle (anterior/left direction) (Fig. 2(b)). This measurement was taken on 11 CT slices (corresponding to ~ 20 mm in the SI direction), beginning with the slice at which the LAD "wrapped" around the anterior edge of the heart, and the subsequent 10 slices moving in the inferior direction, corresponding to a location with high risk of being in the primary field. The minimum distance measured on these slices was then calculated as the minimum distance between the structures. Records of measured BHL at simulator were collected and the correlation between breath-hold level and mid-LAD chest wall separation were investigated to determine if a minimum breath-hold level recommendation for VM-DIBH eligibility could be found.

## III. RESULTS

### A. Patient characteristics

In total 222 cine images from 42 patients were retrospectively evaluated for chest wall positioning measurements. The median age of the patients was 55.5 years with a range of 36–78 years; 31 patients had breast-conserving surgery and 11 had mastectomy. Twenty-four patients were treated with tangents alone, 15 were treated with RNI including IMC nodes, and 3 patients had RNI excluding the IMC nodes. The majority of patients received either 42.5 Gy in 16 fractions (n = 29) or 40 Gy in 16 fractions (n = 10). The remaining patients received 45 Gy in 25 fractions (n = 2) and 50.4 Gy in 28 fractions (n = 1). Due to poor image quality and difficulty identifying the heart on some MV images, only 205 cine images from 40 patients were evaluated for MHD measurements. Complete records of BHL measurements made at simulation were collected for 31 out of 42 patients.

### B. Cine imaging and analysis

#### *B.1 Chest wall position measurements*

As shown in Fig. 3, the majority of fields (80.6%) had maximum DIBH chest wall positioning errors ($D_{SETUP}$) less than 5 mm, and 96.8% of fields had less than 3 mm of motion during beam-on ($D_{BEAM}$). Patients were more likely to have negative intrabeam motion, indicating exhale or relaxation during DIBH (Fig. 3(b)). Just one measured field had > 3 mm of motion in the positive μ direction. Since only one breath-hold was required for each field, intrafraction motion (same fraction, different medial field) was only evaluated for the patients with two medial fields of different energies (n = 13) and was found to have an average of 1.0 mm with range 0.0–3.4 mm.

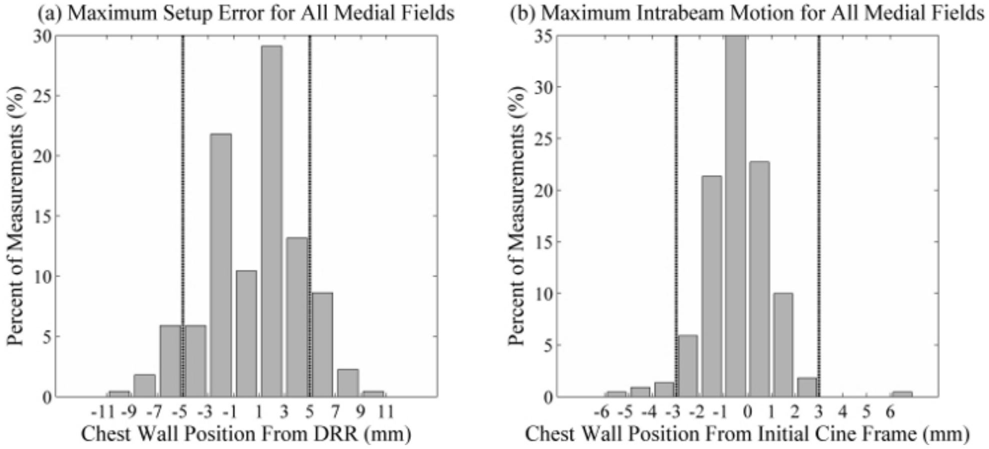

Fig. 3. (a) Distribution of $D_{SETUP}$ measurements describing the maximum setup error for all medial fields. Vertical lines at ± 5 mm indicate imaging setup error tolerance. (b) Distribution of $D_{BEAM}$ measurements describing the maximum intrabeam motion for all medial fields. Vertical lines at ± 3 mm indicate breath-hold stability tolerance.

Table 1 shows the mean of patient means (M); the systematic error (Σ), defined as the standard deviation of the patient means;[17] and the random error (σ), defined as the root mean square of the patient standard deviations,[17] for all patients, and for patients with intact breast versus chest wall. All values of M were less than 1 mm, indicating no large systematic process errors. Intact-breast patients had a larger population systematic error than chest wall patients.

Table 1. Setup uncertainties showing population mean displacement (M), systematic (Σ), and random (σ) setup errors for all patients and separated by surgery type. Wilcoxon Rank-Sum tests were used to determine p-values.

| | *All Patients (n=42)* | *Intact Breast (n=30)* | *Chest Wall (n=12)* | *p-value* |
|---|---|---|---|---|
| M | 0.4 | 0.8 | 0.1 | 0.6 |
| Σ | 3.0 | 3.2 | 2.6 | |
| σ | 2.6 | 2.6 | 2.6 | 0.8 |

### *B.2 Maximum heart distance measurements*

The measured treatment MHDs for all fields, sorted by magnitude, is outlined in Table 2. The clinical significance of irradiating a small portion of the heart/pericardium is not fully understood,[5] as variations in anatomy including the thickness of the pericardial fat pad can alter the perceived heart volume. In this study an arbitrary MHD of 10 mm was selected to separate "smaller" and "larger" MHDs. The average MHD over all measured fields was 2.5 mm (range: 0.0–19.1 mm). The heart was not visible in 150 / 205 measured fields; for the remaining 55 fields where the heart was visible the mean MHD was 9.4 mm (range: 3.3–19.1 mm). When the heart was visible, there were no MHD measurements smaller than 3 mm, potentially representing the lower limits of detection for this methodology. There was no significant difference in treatment MHD for treatment type (standard tangents versus modified wide tangents, $p = 0.11$) or surgery type (mastectomy versus breast-conserving surgery, $p = 0.33$). MHD measurements corresponding to plans where the heart had been planned partially inside the field were significantly higher than those from the heart planned fully out of the field ($p < 0.001$).

Table 2. Maximum heart distance measurements for all patients and separated by planned heart position relative to the field edge (in or out) and tangent type (standard or modified wide). Number of beams (b) and patients (p) for each subgroup in titles. Wilcoxon Rank-Sum tests were used to determine p-values.

| | | *Heart Position in Plan* | | *Tangent Type* | |
|---|---|---|---|---|---|
| *MHD (mm)* | *Total # Beams (b=205; p=40)* | *In Field (b=60; p=12)* | *Out of Field (b=145; p=28)* | *Standard (b=129; p=26)* | *Modified Wide (b=76; p=14)* |
| 0 | 150 (73%) | 28 (47%) | 122 (84%) | 99 (77%) | 51 (67%) |
| 0<MHD<10 | 31 (15%) | 11 (18%) | 20 (14%) | 17 (13%) | 14 (18%) |
| ≥10 | 24 (12%) | 21 (35%) | 3 (2%) | 13 (10%) | 11 (14%) |
| | *Mean MHD (mm)* | 5.9 | 1.1 | 2.1 | 3.3 |
| | *p-value* | <0.001 | | 0.11 | |

Figure 4 shows the planned and average treatment MHDs for each patient. Although 13/40 patients with the heart planned out of the field had some heart visible (MHD > 0) in one or more cine images (Fig. 4(a)), this only corresponded to three beams with MHD > 10 mm (Table 2). All patients with average treatment MHD > 10 mm (n = 5) had a portion of the heart planned in the field; however, all five patients had a smaller planned MHD compared to the average treatment MHD (Fig. 4). The average patient treatment MHD was slightly larger for patients treated with modified wide tangents (4.0 mm) versus standard tangents (2.3 mm); but not significantly so ($p = 0.24$). No correlation was found between chest wall positioning error and MHD differences from planning (data not shown, $R^2 < 0.1$).

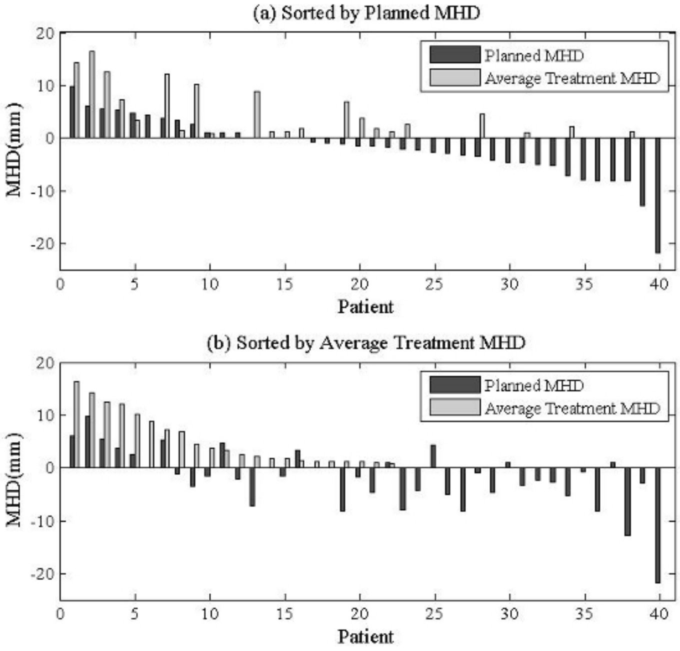

FIG. 4. Planned and treatment MHD measurements (a) sorted by decreasing planned MHD, and (b) sorted by decreasing average MHD during treatment. Planned MHDs were measured on DRRs as the distance from the field edge to the heart contour. Negative numbers indicate the distance that the heart was planned out of the field. The patients with the heart planned in the field were the ones with the highest average treatment MHDs. 18/40 patients had MHD = 0 on all measured fractions.

## C. Correlation between breath-hold level and LAD chest wall separation

The correlation between BHL and mid-LAD chest wall separation was investigated for 31 patients. The LAD wrapped around the heart at a median distance of 24 mm inferior to the top of the heart contour (range: 6–38 mm). As shown in Fig. 5, all patients achieved > 10 mm mid-LAD chest wall separation (median: 20 mm, range: 12–37 mm) with voluntary breath-hold in a region of the heart where the LAD is likely to enter the tangent beams with a large range of measured BHLs (3–21 mm). No correlation was observed between BHL measured at simulator and separation between the mid-LAD and the chest wall.

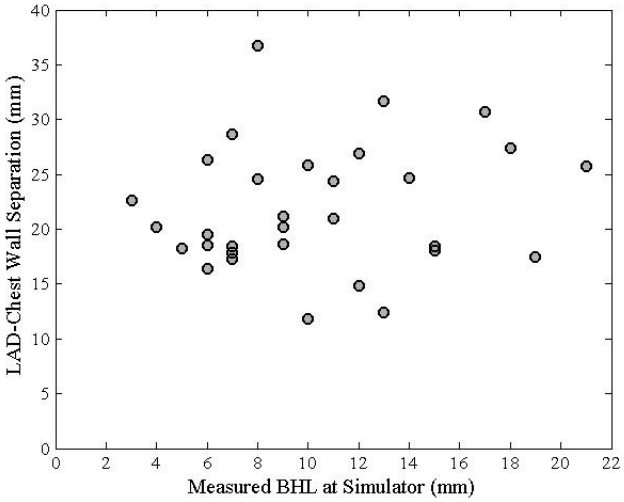

Fig. 5. Separation between the mid-LAD and chest wall versus the measured breath-hold level at simulator for 31 patients. There was no population-based correlation between the distance from the mid-LAD to the chest wall and the measured BHL.

## IV. DISCUSSION

The VM-DIBH technique described herein was successfully implemented for all eligible patients in our clinic. While the results shown here are for wedged tangent fields, we have since switched our clinical practice to a field-in-field technique and found no changes to the VM-DIBH process were necessary. The existing “wide-view” in-room camera configuration at our center was ideal for implementing VM-DIBH; however, centers with narrower camera angles may need to change their camera positioning in order to introduce this technique.

Data were not collected on the percentage of patients with reproducibility issues at simulation or at the treatment unit. Generally, this was not a commonly reported problem, although some patients required additional coaching at simulation to achieve reproducible breath-holds. All patients were instructed to practice DIBH at home between simulation and the first day of treatment. We found this made patients more relaxed and breath-holds more reproducible on the first day of treatment. Large reproducibility issues at simulation could result in increased treatment times on the treatment unit. This issue was addressed on a case-by-case basis, but included further coaching at the treatment unit, increasing scheduled treatment times, and daily imaging to ensure adequate coverage and heart sparing.

The cardiac dose-sparing advantages of VM-DIBH were examined in a previous planning study.[7] Our technique is similar to the voluntary breath-hold (VBH) technique used in the UK HeartSpare study;[13] however, the two techniques were developed independently. Bartlett et al.[13] characterized the VBH technique using electronic portal imaging (EPID) and cone-beam CT. For offline EPID measurements, random and systematic errors of $\sigma = 2.1$ mm and $\Sigma = 1.8$ mm, respectively, were reported in the μ direction of the medial tangent beam. In contrast, we aimed to characterize the worst-case scenario, taking the maximum setup uncertainty measurements from during-treatment cine images on nonimaging days ($D_{SETUP}$). Using central lung distance measurements, we found setup uncertainties of $\sigma = 2.6$ mm and $\Sigma = 3.0$ mm. Overall, patients with intact breast had higher systematic uncertainties than those who underwent mastectomy (3.2 mm versus 2.6 mm). While these uncertainties are larger than some values reported for more resource-intensive methods of DIBH, they are consistent with similar studies of free-breathing breast setup uncertainties without daily imaging.[19] Previous DIBH studies have suggested that daily setup imaging could aid in reducing setup uncertainties.[20] Since implementing the VM-DIBH technique at our center, several refinements to the process described in the methods have been proposed, including using only the lateral line BHL measurement for setup on the treatment unit, performing shifts in DIBH, and immediately measuring couch top to midaxillary distance on the DIBH CT to confirm that the patient was in the measured position during the DIBH CT scan.[13] These changes may reduce the systematic error.

Using the HeartSpare VBH protocol, Colgan et al.[21] recently reported < 1 mm displacement during beam delivery for 93% of patients using offline matching of cine images. We found that 97.3% of beams had breath-hold stability within 3 mm in the μ direction ($D_{BEAM}$), with with 6/222 beams from 4/42 patients falling outside this range. These results are consistent with Jensen et al.,[14] who used a similar in-room, laser-based method and found intrabeam stability to be within 3 mm for 23/27 patients. A limitation of these measurements is that the entire breath-hold could not be monitored due to the entrance of the wedges into the cine images (i.e., for a 20 s tangential field with a 45° wedge, measurements were made only on the first 14 s). This may result in underestimation of intrafraction motion.

To our knowledge, we are the first to characterize in-treatment MHD for a voluntary breath-hold technique. Mean heart shifts of 13 mm between FB and DIBH in the μ direction have been previously reported.[8] Therefore, the expected change in MHD between FB and DIBH is on the order of 10 mm. The clinical implications of a small portion of the heart entering the primary beam as measured on a cine image are unknown;[5] however, we found that larger (> 10 mm) in-treatment MHD measurements were uncommon, and were most likely in patients where a portion of the heart was planned in the field. While the aim of implementing DIBH is to remove the heart from the primary beam, the heart may be planned in the field as a result of a clinical decision by the radiation oncologist to improve target coverage on the chest wall, breast, or IMC nodes. Five out of 40 patients had consistent systematic heart positioning errors resulting in average MHD > 10 mm (Fig. 4). Increased MHD during treatment can occur due to a complicated combination of setup uncertainty and/or breath-hold level errors. To avoid unintentional heart irradiation, the amount of cardiac shielding should reflect the tolerances for these sources of error whenever possible. This could be achieved by using a planning organ-at-risk volume (PRV) margin.[22] Goody et al.[23] conducted a similar MHD study for 128 free-breathing patients. The mean measured MHD in our study was smaller than the FB study (2.5 mm versus 3.9 mm), as was the proportion of patients with the heart planned out of the field where the heart was subsequently visible during at least one treatment field (25% vs. 49.4%). The proportion of patients with > 10 mm average treatment MHD was comparable between the two studies (12% vs. 11%).

In this study we do not present estimations of cardiac dose, but instead focus on cardiac positioning. While the relationship between MHD and cardiac dose has been characterized for open treatment fields,[24] fields with cardiac shielding are more complex, as the location and volume of the heart in the field will influence the cardiac dose. MHD measurements on the DRRs were consistently smaller than in the cine images due to known differences between heart identification in projected CT contours and the pericardial shadow on MV portal images.[8] For this reason, planned and treatment MHDs were not directly compared, except in examining the correlation between CW positioning errors and MHD differences from planning. We found that chest wall displacement from the planned position was not predictive of increased MHD during treatment.

We are the first to demonstrate the use of a low-resource DIBH technique using a modified wide tangent for IMC nodal irradiation, where the tangent fields were widened to include the IMCs and narrowed inferiorly to reduce lung and cardiac doses.(15) The shape of the modified wide tangent fields could increase the chance of unintended cardiac irradiation with incorrect positioning; however, we found no significant difference in chest wall positioning uncertainty or MHD measurements between standard and modified wide tangent beams. RNI treatments also typically include supraclavicular fields; however, field matching uncertainty during VM-DIBH was not evaluated here.

We observed no correlation between BHL and mid-LAD chest wall separation; therefore, no evidence for minimum BHL eligibility was found. This result is likely due to differences in patient anatomy, and suggests that most patients are able to achieve heart displacement for cardiac sparing with a voluntary breath-hold regardless of the measured BHL.

## V. CONCLUSIONS

We successfully implemented and characterized a low-resource visually monitored DIBH technique for left-sided breast cancer patients. The positional accuracy of this technique is comparable to other low-resource techniques in terms of chest wall positioning and stability. VM-DIBH requires very little change in clinical workflow, and is less costly and resource-intensive than many of the commercially available DIBH monitoring techniques. We found no evidence to support a minimum BHL for VM-DIBH eligibility, indicating that all patients who can tolerate voluntary DIBH may yield benefit from it in terms of cardiac positioning.

## ACKNOWLEDGMENTS

The authors wish to thank Roseanne Moore and Coline Dirkse for their contributions in developing the VM-DIBH process, Tracey Lundstrom and Gillian Ecclestone for their help with describing the clinical procedure, and Alexandra Guebert for her assistance with data collection.

## COPYRIGHT

This work is licensed under a Creative Commons Attribution 3.0 Unported License.

## Supporting information

Supplementary Material

Supplementary Material
