# Supplementary material for: Evaluation of target and cardiac position during visually monitored deep inspiration breath‐hold for breast radiotherapy
Source: J Appl Clin Med Phys. 2016 Jul 8;17(4):25–36. doi: 10.1120/jacmp.v17i4.6188 (PMC5690055; doi:10.1120/jacmp.v17i4.6188)
Supplement: Supplementary file 1 — Supplementary Material [file ACM2-17-025-s001.pdf]

Retrospective evaluation of visually monitored deep inspiration breath hold for breast cancer patients using edge detection

L. Conroy[1,2], R. Yeung[3], S. Quirk[1,2], T. Phan[3], and W.L. Smith[1,2,3]

[1] Tom Baker Cancer Centre/Department of Medical Physics, Calgary, Canada
[2] University of Calgary/Department of Physics and Astronomy, Calgary, Canada
[3] University of Calgary/Department of Oncology, Calgary, Canada

Abstract— **Purpose: Deep inspiration breath hold (DIBH) can reduce cardiac dose during left-sided breast cancer radiotherapy. This study uses cine imaging with edge detection to evaluate a visually-monitored DIBH technique (VM-DIBH).**

Methods: Cine images were acquired weekly during the medial tangent field of patients treated with VM-DIBH. Edge detection was used to identify the field borders and chest wall edges in digitally reconstructed radiographs (DRRs) and cine images of 15 patients. The distance between the field border and chest wall was measured at the geometric center of the field and used as a surrogate for patient position during breath hold. Setup uncertainties were found by comparing DRR measurements to the first cine image measurement for each fraction. Intra-beam motion during individual breath holds was assessed by comparing the first cine image measurement to all subsequent cine image measurements for each fraction.

Results: The mean setup uncertainty (M) was 1.2 mm; random (σ) and systematic (Σ) setup errors were both 2.0 mm. The chest wall position was within 5 mm of the DRR position in 92% of cine images. Intra-beam motion was within +/- 2 mm in 98% of images, and was slightly skewed in the posterior direction, indicating that patients tend to relax or exhale during breath hold.

Conclusion: Edge detection of field borders and the chest wall in cine images and DRRs was successfully used to evaluate inter-fraction and intra-beam uncertainties for a VM-DIBH technique. Setup uncertainties and chest wall position measurements indicated adequate breath hold setup reproducibility for the majority of patients. Intra-beam motion measurements showed excellent stability of breath hold during treatment.

Keywords— **Breast Cancer, Motion Management, Portal Imaging, Edge Detection, Setup Uncertainties**

I. Introduction

Breast cancer is commonly treated with surgery followed by radiation therapy (RT) of the breast and/or chest wall and involved lymph nodes. Although RT reduces the risk of local recurrence in breast cancer patients, it also results in increased risk of heart disease, particularly when delivered to the left breast [1]. Delivery of breast RT with deep inspiration breath hold (DIBH) reduces this risk by decreasing dose delivered to the heart. Methods for monitoring breast DIBH include active breathing control (ABC) [2], real-time imaging [3], surface imaging [4] and other non-commercial techniques [2, 5]. At our center, we use a non-commercial visually monitored technique (VM-DIBH). In this paper we demonstrate a semi-automatic method to retrospectively evaluate intra-beam and inter-fraction variation in chest wall position during DIBH treatment using cine electronic portal imaging (EPID) and digitally reconstructed radiograph (DRR) images with edge detection. The results are presented for a subset of left breast cancer patients treated using the VM-DIBH technique.

II. Materials and Methods

A. Patient Data

The study population consists of 15 patients with left sided breast cancer treated with VM-DIBH radiation therapy at our institution between March and August 2013. Cine EPID images were taken at least once weekly during the delivery of medial tangent fields using an AS1000 EPID (Varian Medical Systems, Inc.) in half resolution mode.

B. Simulation

Left-sided breast cancer patients able to maintain DIBH for 20 seconds or longer were included in this study. Patient simulations and treatments were performed in the supine position with wing-board immobilization. During simulation, the distance from the couch top to the mid-axillary line was measured and recorded for free-breathing (FB) and DIBH. DIBH reproducibility was established by repeating the measurement at least three times. The CT scan was taken with monitoring bellows to ensure DIBH stability throughout the scan.

C. Treatment Planning

3D-CRT treatment plans were developed on the DIBH CT scan. Tangential enhanced dynamic wedge fields were

© Springer International Publishing Switzerland 2015

D.A. Jaffray (ed.), *World Congress on Medical Physics and Biomedical Engineering, June 7-12, 2015, Toronto, Canada*,
IFMBE Proceedings 51, DOI: 10.1007/978-3-319-19387-8_143

defined to encompass the whole breast or chest wall target using anatomical landmarks. Wide tangents were used for patients receiving internal mammary chain (IMC) irradiation and a 3- or 4-field technique was used for axillary/supraclavicular node irradiation. Patients receiving boost were included in the study; however no cine images were collected for boost fractions.

D. Radiotherapy Treatment and Imaging

After patients were set up for treatment in FB the displacement from the couch top to the mid-axillary lateral tattoo was recorded and compared to the simulation measurement. Patients were then instructed to take a deep breath and hold, and the distance from the couch top to mid-axillary line was measured in DIBH. This was repeated until the displacement between FB and DIBH was the same as recorded during simulation. A line was drawn on the left lateral side of the patient where the in-room lasers fell during DIBH. In the control room, therapists focused the treatment room cameras to the lines on the patient for DIBH monitoring. Once ready to begin treatment, the patient was asked to perform a breath hold, and the therapist turned on the beam when the line was coincident with the in-room lasers. Treatment was interrupted if the line moved away from the lasers. As per clinical protocol, EPID image guided setup was performed on the first three fractions and once weekly afterward. Shifts were applied if setup differences were > 5 mm. Cine images of the medial tangent field were acquired at least once per week on non-imaging days for retrospective evaluation only.

E. Image Processing

After all patients were treated, the medial tangent field DRRs from the simulation scans and the cine images of the medial tangent fields were exported from the treatment planning system. Image analysis was performed in MATLAB (MATLAB 7.9.0, The MathWorks Inc., Natick, MA).

Simulation DRRs: Planned field edges were extracted from the DICOM header information and applied as a mask to the DRR images to produce a beam's eye view of the planned left-medial tangent field (Figure 1a). A Canny edge-detection filter was applied to detect the position of the chest wall in the DRR images. The distance between the left field border and the chest wall was measured at the geometric center of the field (D_{DRR}). When the center of the field coincided with a multileaf collimator (MLC) leaf edge, the measurement was taken 3 mm below the edge in both the DRR and cine images.

a.

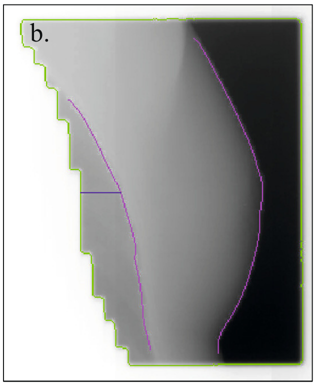

Fig. 1 Chest wall edge detection of field borders (green) and chest wall (pink) as well as chest wall position measurement (blue) for a DRR (a) and a corresponding cine image (b).

Treatment cines: Frames containing wedges were automatically identified and removed from the analysis. Canny edge-detection filters were applied to detect the position of the field edges and chest wall in each cine image, *i*. The distance between the left image border and the chest wall ($D_{cine,i}$) was measured at the same field location as the corresponding DRR (Figure 1b). Cine measurements were removed if the chest wall measurement varied from the median beam value by ≥ 1.2 cm, as this level of undetected motion within a breath hold was deemed unlikely. Discarded measurements were further reviewed to confirm that they were a result of failure in the edge detection algorithm.

F. Data Analysis

Chest wall setup uncertainty was measured for each fraction of every patient by subtracting the DRR chest wall measurement from the measurement of the first image of the corresponding cines ($D_{cine,\ 1}$ - D_{DRR}). From these measurements the population mean (M) as well as systematic (Σ) and random (σ) setup uncertainties were computed [6].

The *total chest wall position uncertainty* over all patients, fractions, and cine images was computed by subtracting DRR chest wall measurement from all corresponding cine chest wall measurements ($D_{cine,\ i}$ - D_{DRR}).

Intra-beam chest wall motion was measured for each fraction of each patient by subtracting the first cine chest wall measurement ($D_{cine,1}$) from each of the subsequent frame measurements ($D_{Cine,\ i\ (i\neq1)}$).

III. Results

In total, 1231 Cine images acquired over 86 treatment fractions were analyzed along with 15 DRRs. 12/15 patients

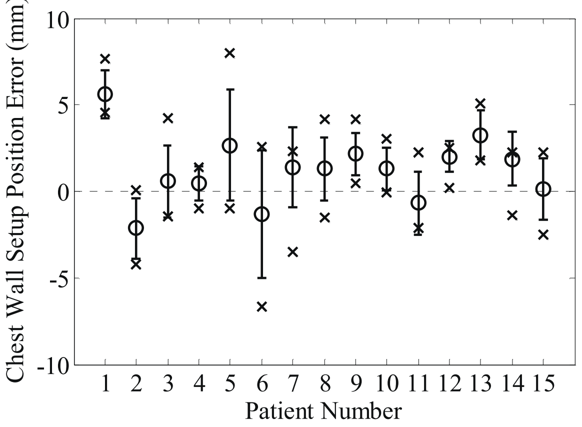

Fig. 2 Chest wall setup uncertainty ($D_{cine,\,1}$ - D_{DRR}). Cine images were taken on 4 – 6 fractions for each patient. The median (circles), standard deviation (error bars), maximum and minimum (crosses) measurements are shown for each patient.

had 4 fractions with cine imaging; the remainder had 5 fractions (1 patient) or 6 fractions (2 patients). The chest wall setup uncertainty median, standard deviation, maximum, and minimum for each patient are shown in Figure 2.

The calculated population mean setup uncertainty (M) was equal to 1.2 mm, and the random (σ) and systematic (Σ) setup uncertainties were both 2.0 mm. Figure 3 shows the distribution of the total chest wall uncertainties. Greater than 92% of all cine chest wall measurements were within +/- 5 mm of the corresponding DRR measurement. The population mean (median) uncertainty over all cine measurements ($D_{cine,\,i}$ - D_{DRR}) was 0.5 mm (0.6 mm), with a standard deviation of 2.8 mm. Maximum and minimum chest wall position uncertainties were 8.5 mm and -9.1 mm.

Figure 4 shows the intra-beam motion measurements over all patients, fractions, and images. 98% of all intra-beam measurements were within +/- 2 mm of the first cine

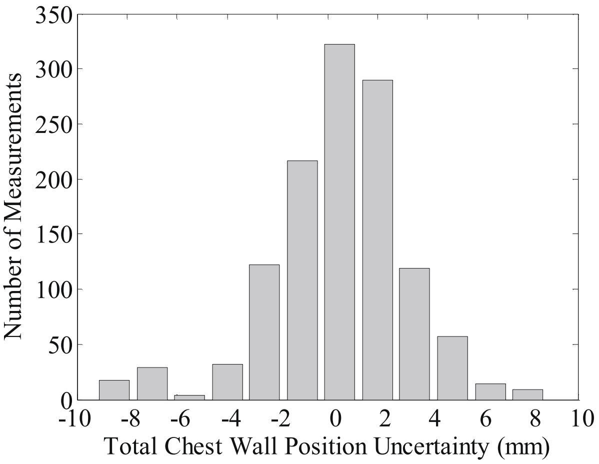

Fig. 3 Total chest wall position uncertainty measurements ($D_{cine,\,i}$ - D_{DRR}) for all patients, fractions, and cine images. The measured chest wall position was within 5 mm of the DRR in 92% of images.

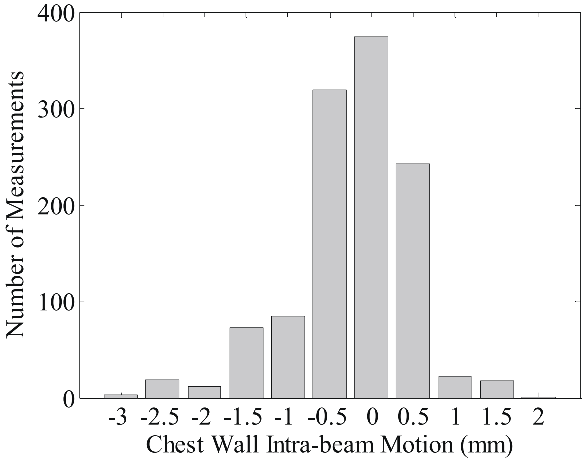

Fig. 4 Intra-beam chest wall motion ($D_{cine,\,i\,(i\neq1)}$ - $D_{cine,1}$) for all patients, fractions, and cine images. All patients moved less than 3 mm during cine acquisition. There is a slight skew in the distribution, indicating that patients may exhale or relax slightly during breath hold.

image. The population mean (median) intra-beam uncertainty ($D_{Cine,\,i\,(i\neq1)}$ - $D_{cine,1}$) was -0.2 mm (0.0 mm) with standard deviation of 0.7 mm. Maximum and minimum intra-beam chest wall motion was 1.9 mm and -2.9 mm. The distribution was slightly skewed to the negative side; this illustrates the tendency for patients to exhale or relax during DIBH.

IV. DISCUSSION

We have demonstrated a method to retrospectively evaluate intra-beam and inter-fraction variation in chest wall position during a visually monitored DIBH technique using DRRs and cine EPID images. Visually monitored techniques are less resource-intensive than other commercially available methods, and do not deliver any additional dose.

Jensen *et al.* used a similar measurement technique with cine measurements of the distance from the field edge to the chest wall for intra-beam motion characterization of two non-commercial DIBH techniques [5]. For their in-room laser VM-DIBH technique, they found the same median intra-beam motion of 0.0 mm, and a slight skew in the distribution towards exhalation, similar to the results shown in Figure 4. It should be noted that all intra-beam motion in our study was measured prior to the wedges due to the limitations of cine imaging. Similarly, Jensen *et al.* only measured intra-beam motion on jaw-collimated open fields, excluding MLC segments [5]. Both sets of measurements may underestimate intra-beam motion, especially for patients who tend to exhale during breath hold.

Bartlett *et al*. characterized their non-commercial VM-DIBH technique with daily EPID imaging and CBCT [2]. Setup uncertainties were measured offline for all fractions, and intra-beam motion was not measured. For the right anterior oblique field, they found a population mean of M = -0.2 mm, and random and systematic setup uncertainties of $\sigma = 2.1$ mm and $\Sigma = 1.8$ mm.

Borst *et al*. used an imaging technique developed in-house to monitor and evaluate DIBH in breast [3]. They found setup uncertainties of $\sigma = 2.8$ mm and $\Sigma = 3.7$ mm (pre-correction) and $\sigma = 0.9$ mm and $\Sigma = 2.0$ mm (post-correction) using real-time in-house EPID and CBCT imaging [3]. Similarly, Remouchamps *et al.* used EPID imaging to quantify setup uncertainties for DIBH monitored using magnetic sensors and found $\sigma = 2.4$ mm and $\Sigma = 1.7$ mm [7]. Our calculated VM-DIBH random and systematic setup uncertainties are comparable to these more resource-intensive techniques.

Typically, a population setup uncertainty mean (M) close to 0 mm is expected; significant deviations from this suggest a potential systematic error in the treatment delivery process. However, our population mean of 1.2 mm is comparable in magnitude to the pre-correction values found in Borst *et al.* [3], where the authors showed that the population mean setup uncertainty can be reduced to M = 0.3 mm with daily imaging. The results presented here are representative of our clinic's practice of imaging for only the first three fractions of treatment and once weekly thereafter.

A subset of patients for which automated edge detection was successful in both the DRR image and the majority of cine images were included in this study. Differences in patient anatomy and image quality impeded robust fully-automated edge detection. Further optimization of this technique in future may allow inclusion of more patient data for a more rigorous characterization with less influence from outlying patients.

Chest wall position uncertainty and setup uncertainty measurements were calculated from measurements made on two different image types (DRRs and cines) using different edge detection filter settings. This may have introduced small systematic measurement errors, for example differences in localization errors due to Gaussian blurring kernels in Canny edge detection. Our results agree well with similar studies in the literature and we estimate this error to be small. Intra-beam motion measurements do not have this systematic error, as offsets from the true edge would be similar in all images.

V. Conclusions

Edge detection of field borders and the chest wall in cine EPID images and DRRs was successfully used to quantify setup uncertainties, chest wall position uncertainties, and intra-beam motion for 15 left-sided breast cancer patients treated with visually monitored DIBH radiotherapy. This low-infrastructure DIBH technique has comparable inter-fraction and intra-beam uncertainties as other more resource-intensive DIBH monitoring techniques.

Acknowledgments

The authors wish to thank Karen Long for her assistance in the collection and organization of patient data for this study.

Conflict of Interest

The authors declare that they have no conflict of interest.

References

1. Darby S, Ewertz M, McGale P et al. (2013) Risk of ischemic heart disease in women after radiotherapy for breast cancer. N Engl J Med 368:987-998.
2. Bartlett F, Colgan R, Carr K et al. (2013) The UK HeartSpare Study: Randomised evaluation of voluntary deep-inspiration breath-hold in women undergoing breast radiotherapy. Radiother Oncol 108:242-247.
3. Borst G, Sonke J-J, den Hollander S et al. (2010) Clinical results of image-guided deep inspiration breath hold breast irradiation. Int J Radiat Oncol Biol 78:1345-1351.
4. Betgen A, Alderliesten T, Sonke J-J et al. (2013) Assessment of set-up variability during deep inspiration breath hold radiotherapy for breast cancer patients by 3D-surface imaging. Radiother Oncol 106:225-230.
5. Jensen C, Urribarri J, Cail D et al. Cine EPID evaluation of two non-commercial techniques for DIBH. Med Phys 41:021730.
6. van Herk M (2004) Errors and margins in radiotherapy. Semin Radiat Oncol 14:52-64.
7. Remouchamps V, Huyskens D, Mertens I et al. (2007) The use of magnetic sensors to monitor moderate deep inspiration breath hold during breast irradiation with dynamic MLC compensators. Radiother Oncol 82:341-348.

Author: Leigh Conroy
Institute: Tom Baker Cancer Centre
Street: 1331 29 St. NW
City: Calgary
Country: Canada
Email: laconroy@ucalgary.ca
