# Supplementary material for: Evaluation of target and cardiac position during visually monitored deep inspiration breath‐hold for breast radiotherapy
Source: J Appl Clin Med Phys. 2016 Jul 8;17(4):25–36. doi: 10.1120/jacmp.v17i4.6188 (PMC5690055; doi:10.1120/jacmp.v17i4.6188)
Supplement: Supplementary file 2 — Supplementary Material [file ACM2-17-025-s002.pdf]

2051

Visually Monitored Deep Inspiration Breath Hold Technique in Left-Sided Breast Cancer Patients Treated With Adjuvant Radiation Therapy: Reproducibility and Reliability

R. Yeung, L. Conroy, K. Long, W. Smith, A. Hudson, R. Moore, C. Dirkse, and T. Phan*; Tom Baker Cancer Centre, Calgary, AB, Canada*

Purpose/Objective(s): Deep inspiration breath hold (DIBH) techniques have shown dose reduction to the heart, left anterior descending and lung in adjuvant radiation therapy (RT) of left-sided breast cancers. Commercial systems for ensuring maintenance and reproducibility of DIBH, such as active breathing control (ABC) and Real-time Position Management (RPM) are not readily available across cancer centers. Visually monitored DIBH technique using coincident in-room lasers and skin marks, without RPM or ABC, may be a feasible alternative. The objective of this study was to evaluate maintenance and inter-fraction reproducibility of a visually monitored DIBH technique for left-sided breast RT using cine images.

Materials/Methods: Twelve left-sided breast cancer patients receiving adjuvant RT were included. Breath hold (BH) was monitored using cine images obtained during delivery of the medial breast tangent fields at least once a week. Chest wall position in the anterior-posterior (AP) and superior-inferior (SI) direction relative to a fixed point at field edge was measured every 2 seconds for each treatment fraction where cine images were acquired. Change in chest wall position during treatment was used to determine maintenance of BH. Inter-fraction reproducibility was determined by measuring change in chest wall position over all treatment fractions for which cine images were obtained. Comparison in chest wall position on the planning digital reconstructed radiograph (DRR) and cine images was used to determine reproducibility of DIBH at simulation and treatment.

Results: At least three cine images were acquired for each patient. Median treatment time for the medial tangential field was 10.8 s (5.0-15.3 s). Chest wall displacement during treatment of all patients was < 0.3 cm. Median intra-beam chest motion was 0.12 cm in AP (0.03-0.29 cm) and 0.08 cm in SI (0.03-0.25 cm) directions. Median change in chest wall position between treatment fractions was greater than intra-beam chest motion (0.45 cm AP, 0.24-1.07 cm and 0.16 cm SI, 0.08-0.25 cm). Chest wall position during treatment was within +/-0.5 cm of the DRR 99% of the time.

Conclusions: Visually monitored DIBH technique for left-sided breast RT demonstrates stability in maintenance of BH for all patients (<0.3 cm chest wall displacement). Although inter-fractional variability in BH was greater than intra-beam variation, chest wall position during treatment was within 0.5 cm of the planning DRR 99% of the time, indicating good reliability of BH during and between treatment fractions. Reproducibility of set-up between simulation and treatment is also within standard range. Prospective cohorts comparing cine images with RPM measurements are in progress to validate our results.

Author Disclosure: **R. Yeung:** None. **L. Conroy:** None. **K. Long:** None. **W. Smith:** None. **A. Hudson:** None. **R. Moore:** None. **C. Dirkse:** None. **T. Phan:** None.

2052

Development of a Photonumeric Scale for Acute Radiation Dermatitis in Breast Cancer Patients

D. Shumway,[1] N. Kapdia,[2] T. Do,[1] K. Griffith,[1] M. Feng,[1] R. Jagsi,[1] Y. Helfrich,[1] A. Liss,[1] E. Gillespie,[1] A. Miller,[1] and L. Pierce[1]; *[1]University of Michigan, Ann Arbor, MI, [2]Dartmouth Hitchcock Medical Center, Lebanon, NH*

Purpose/Objective(s): Scales for rating acute radiation dermatitis are inconsistent and have not been validated despite decades of clinical use. We sought to design a photonumeric scale to reliably describe acute radiation dermatitis in breast cancer patients undergoing RT.

Materials/Methods: Patients undergoing RT for breast cancer were enrolled on a prospective study that included photographs and the reporting of physician-rated erythema at baseline and 2, 4, and 6 weeks after initiating RT. Erythema was also quantified using a hand-held colorimetric device. Photographs were taken using a standardized protocol that included 3 views to fully assess the breast/chest wall, axilla, and inframammary folds. Patients reported their Fitzpatrick skin type using a questionnaire as a putative measure of baseline skin pigmentation. Two hundred nine photographs from 35 patients were clustered according to the apparent severity of acute radiation dermatitis and a descriptive photonumeric scale was developed via an iterative process until group consensus of radiation oncologists and dermatologists was achieved. Two raters with experience in the evaluation of acute radiation dermatitis and treatment of breast cancer patients used the photonumeric scale to independently score the entire collection of photographs. Intra- and inter-rater agreements were assessed using weighted kappa scores.

Results: Of the 35 patients enrolled, 20% experienced severe erythema, and 40% experienced dry or moist desquamation. Using the photonumeric scale we observed high intra-rater agreement for independent ratings of erythema (79 to 89% agreement fraction, kappa 0.65 to 0.81) and desquamation (79 to 80% agreement fraction, kappa 0.53 to 0.6). Similarly, we observed high inter-rater agreement for independent ratings of erythema (76% agreement fraction, kappa 0.62) and desquamation (80% agreement fraction, kappa 0.53). Quantitative measurements of erythema using colorimetry correlated strongly with photonumeric grade (correlation coefficient 0.76, $p < 0.001$), as did physician-rated erythema at the point-of-care ($p < 0.001$). Fitzpatrick score was not significantly associated with maximum photonumeric erythema grade ($p = 0.14$).

Conclusions: These results report the utility of a new photonumeric scale with high intra- and inter-rater reliability for acute radiation dermatitis in breast cancer patients. Use of colorimetric measurements added little above what was achieved using the scale. The photonumeric scale will facilitate consistent reporting of acute radiation dermatitis in research and clinical settings using a simple, standardized instrument. Future work will include validation in a larger data set and correlation with patient-reported outcomes.

Acknowledgement: This research was supported by funding provided by a Munn Idea Grant (G011480).

Author Disclosure: **D. Shumway:** None. **N. Kapdia:** None. **T. Do:** None. **K. Griffith:** None. **M. Feng:** None. **R. Jagsi:** A. Employee; University of Michigan. E. Research Grant; NIH, American Cancer Society, Breast Cancer Research Foundation, National Comprehensive Cancer Network. J. In-kind Donation; Provision of drug only from Abbvie Pharmaceuticals for a Phase I trial (trial itself investigator-initiated and funded by BCRF not industry). K. Advisory Board; Eviti Medical Advisory Board. S. Leadership; Chair ROI Research Committee, Chair ASCO Ethics Committee. **Y. Helfrich:** G. Consultant; Galderma. **A. Liss:** None. **E. Gillespie:** None. **A. Miller:** None. **L. Pierce:** None.

2053

Regional Nodal Radiation Therapy in Breast Cancer Patients With Positive Nodes Who Convert to Negative Nodes After Neoadjuvant Chemotherapy

M. Yassa, Z. Fawaz, D. Nguyen, B. Fortin, L. Sideris, and P. Vavassis; *Hopital Maisonneuve-Rosemont, Montreal, QC, Canada*

Purpose/Objective(s): Evidence from the NSABP B-18 and B-27 trials has shown that patients with locally advanced breast cancer presenting a complete response (CR) after undergoing neoadjuvant chemotherapy (neoCT) have an improved prognosis. However these studies were conducted during the previous era of radiation therapy (RT) techniques and chemotherapy regimens. Regional RT was usually not administered in these trials. The aim of this study was to assess outcomes in patients based on nodal response after neoCT in a modern cohort of patients with locally advanced breast cancer presenting with positive axillary nodes (cN+).

Materials/Methods: In this single institution retrospective review, we identified all patients with locally advanced breast cancer presenting with positive axillary nodes treated between January 2003 and December 2010 using neoCT.

Results: One hundrd ninety-seven women with cN+ locally advanced breast cancer were identified. Mean age at diagnosis was 52 years old; 88%
